# Targeting EZH2 as a novel therapeutic strategy for sorafenib‐resistant thyroid carcinoma

**DOI:** 10.1111/jcmm.14365

**Published:** 2019-05-13

**Authors:** Zhengshi Wang, Jiaqi Dai, Jie Yan, Yun Zhang, Zhiqiang Yin

**Affiliations:** ^1^ Thyroid Center Shanghai Tenth People's Hospital Tongji University School of Medicine Shanghai Center for Thyroid Diseases Shanghai China

**Keywords:** EZH2, H3K27, miR‐124/506, Sorafenib, thyroid carcinoma

## Abstract

Thyroid carcinoma is the most common endocrine malignancy. Surgery, post‐operative selective iodine‐131 and thyroid hormone suppression were the most common methods for the therapy of thyroid carcinoma. Although most patients with differentiated thyroid carcinoma (DTC) showed positive response for these therapeutic methods, some patients still have to face the radioactive iodine (RAI)‐refractory problems. Sorafenib is an oral multikinase inhibitor for patients with advanced RAI refractory DTC. However, the side effects and drug resistance of sorafenib suggest us to develop novel drugs and strategies for the therapy of thyroid carcinoma. In this study, we firstly found that patients with sorafenib resistance showed no significant change in rapidly accelerated fibrosarcoma and VEGFR expression levels compared with sorafenib sensitive patients. Moreover, a further miRNAs screen by qRT‐PCR indicated that miR‐124‐3p and miR‐506‐3p (miR‐124/506) were remarkably reduced in sorafenib insensitive patients. With a bioinformatics prediction and functional assay validation, we revealed that enhancer of zeste homolog 2 (EZH2) was the direct target for miR‐124/506. Interestingly, we finally proved that the sorafenib resistant cells regained sensitivity for sorafenib by EZH2 intervention with miR‐124/506 overexpression or EZH2 inhibitor treatment in vitro and in vivo, which will lead to the decreased tri‐methylation at lysine 27 of histone H3 (H3K27me3) and increased acetylated lysine 27 of histone H3 (H3K27ac) levels. Therefore, we conclude that the suppression of EZH2 represents a potential target for thyroid carcinoma therapy.

## 1 INTRODUCTION

Thyroid carcinoma is the most common endocrine malignancy, accounting for about 1% of all systemic malignancies.^1^, ^2^, ^3^ It is estimated 35 000 patients die of thyroid carcinoma with more than 213 000 new cases of thyroid carcinoma are arose every year worldwide.^4^, ^5^ Approximately 94% of all thyroid cancers are differentiated thyroid cancer, including three subtypes of papillary, follicular and poorly differentiated thyroid cancers.^6^ Although most of the patients with differentiated thyroid carcinoma have demonstrated a good therapeutic response and prognosis after the therapy of surgery, post‐operative selective iodine‐131 and thyroid hormone suppression, some patients still have radioactive iodine (RAI)‐refractory problems during the process of the disease or after treatment.^7^ The survival rate of the patients with locally advanced or metastatic RAI‐refractory differentiated thyroid cancer is significantly reduced, which is the focus and difficulty in clinical diagnosis and treatment and needs to be further explored.^7^, ^8^

Sorafenib, an oral multikinase inhibitor, was initially developed to target rapidly accelerated fibrosarcoma (RAF) kinase in the MAPK pathway.^9^ Additionally, sorafenib was found to have potent inhibitory roles of RTKs, VEGFR and RET kinases, leading to the approval of the United States Food and Drug Administration in November 2013 for patients with advanced RAI therapy refractory differentiated thyroid carcinoma (DTC).^9^, ^10^, ^11^, ^12^ Considering the side effects, cost and lack of overall survival benefit of sorafenib during the treatment of metastatic DTC patients, it is essential and urgent to develop novel drugs and strategies for the therapy of thyroid carcinoma.^9^, ^13^

The dysregulation of microRNA (miRNA) expression is involved in variety of human malignancies and emerging miRNAs have been demonstrated playing important roles during the tumorigenesis of thyroid carcinoma, including miR‐199b‐5p,^14^ miR‐214,^15^ miR146b‐5p,^16^ miR‐129^17^; miR‐124‐3p^18^; miR‐506‐3p^19^; miR‐150‐5p 20 and miR‐205.^21^ Enhancer of zeste homolog 2 (EZH2), a subunit of polycomb repressor complex 2 (PRC2), is important for the gene transcriptional regulation through H3K27me3.^22^, ^23^ Many studies have revealed the inappropriate expression of EZH2 in various aggressive cancers, including thyroid carcinoma.^24^, ^25^ However, there is currently no information on the relationship between EZH2 expression and the cancer therapy.

In this study, we firstly focused on the gene expression profile between the sorafenib sensitive and resistant tumour tissues collected from 16 patients with thyroid carcinoma. Then, tumour cell lines were established from sorafenib sensitive and resistant thyroid carcinoma tissues to further confirm the resistance of sorafenib in vitro. To reveal the detailed molecular mechanism for the resistance of sorafenib in the sorafenib resistant patients, we also investigated the differential expressed miRNAs and their target genes between the sorafenib sensitive and resistant tumour tissues. Most importantly, we aim to discover viable therapeutic strategy to be offered to patients with thyroid cancer to decrease tumour burden and progression.

## 2 MATERIALS AND METHODS

### 2.1 Clinical samples collection

A total of 16 tumour tissues from patients with thyroid carcinoma used in this study were collected from the Shanghai Tenth People's Hospital, Tongji University School of Medicine hospital. Informed consent was obtained from the patient and all the tissue collection procedure was approved by Shanghai Tenth People's Hospital, Tongji University School of Medicine committee. Samples were put immediately into the pre‐cold cell culture medium after surgery. Tissues will be divided into three parts for the RNA and protein extraction and the establishment of patients‐derived cell lines.

### 2.2 The establishment of patients‐derived cell lines

Tumour tissues collected from clinic were subsequently rinsed in PBS containing penicillin and streptomycin and cut into small pieces. The tumour fragments were then digested with 1 mg/mL collagenase, 0.02 mg/mL DNAase and 0.01 mg/mL hyaluronidase for 16‐20 hours at room temperature. Then, the specimens were cultured using DMEM/F12 medium containing 10% FBS, thyroid stimulating hormone, triiodothyronine (T3), dermal growth factor (EGF) and hydrocortisone. The tissues were then kept at 37°C in a humidified atmosphere with 5% CO_2_ and the patients‐derived cell lines will be established a few days later.

### 2.3 Reagents and antibodies

DMEM/F12K, trypsin, penicillin and streptomycin were purchased from Life technology. Sorafenib was purchased from Sigma (St. Louis, MO, USA). The EZH2 inhibitor EPZ‐6438 was obtained from Adooq Bioscience (Shanghai, China). Antibodies against RAF and VEGFP were brought from Abcam (Cambridge, MA, USA). The anti‐GAPDH and anti‐H3 antibodies were obtained from Sigma. Antibodies used to detect the protein levels of EZH2, H3K27me3 and H3K27Ac were purchased from Abcam. The secondary antibodies for Western blot were purchased from Jackson ImmunoReseach (West Grove, PA).

### 2.4 The miRNA mimics and transfection

The miR‐124/506 mimics and the control (NC) sequence were synthesis in Shanghai Liangtai Biotechnology (Shanghai, China). Cells were seeded into 60 mm dish and the mimics were transfected into the cells 24 hours later with Lipofectamine^®^ 2000 Reagent (Life Technologies, Pleasanton, CA) according to the manufacture's protocol.

### 2.5 Total RNA extraction and qRT‐PCR procedure

The total RNA from tumour tissues and cells were extracted with TRIzol reagent (Invitrogen, Waltham, MA) according to manufacturer's instructions. The quantitative real time PCR (qRT‐PCR) was performed with SuperReal PreMix Plus (TIANGEN Biotech, Beijing, China), followed by a RNA reverse transcription performed by a Quantscript RT Kit (TIANGEN Biotech). The expression levels of miRNAs were presented as relative values to U6, an internal control. The mRNA levels of these genes were presented as relative values to GAPDH, an internal control. All the experiments were performed in triplicate.

PCR primers: RAF: forward primer: CTTCCCCAGACCGCGATTC, reverse primer: CGACCACCTCTATGGTGACCT, VEGFR: forward primer: GAGGAGCAGTTACGGTCTGTG, reverse primer: TCCTTTCCTTAGCTGACACTTGT, hsa‐miR‐199b‐5p: forward primer: CAGCCCAGTGTTTAGACTATC, reverse primer: GTCCAGTTTTTTTTTTTTTTTGAACAG, hsa‐mir‐214: forward primer: GACAGAGTTGTCATGTGTCTGCCTGTCTACACTTGCTGTGCAGAACATCCGCTCAC, reverse primer: GTCCAGTTTTTTTTTTTTTTTAGGCT, hsa‐miR‐146b‐5p: forward primer: GCAGTGAGAACTGAATTCCA, reverse primer: CCAGTTTTTTTTTTTTTTTCAGCCT, hsa‐mir‐129: forward primer: GCGGTCTGGGCTTGCTGTTCCTCTCAACAGTAGTCAGGAAGCCCTTAC, reverse primer: GGTCCAGTTTTTTTTTTTTTTTAGATAC, hsa‐miR‐124‐3p: forward primer: AGGCACGCGGTGA, reverse primer: TCCAGTTTTTTTTTTTTTTTGGCA, hsa‐miR‐506‐3p: forward primer: GCAGTAAGGCACCCTTCT, reverse primer: GTCCAGTTTTTTTTTTTTTTTCTACTCA, hsa‐mir‐205: forward primer: AGACAATCCATGTGCTTCTCTTGTCCTTCATTCCACCGGAGTCTGTCTCATACCCAACCAGATTTCAGTGGAGT, reverse primer: GGTCCAGTTTTTTTTTTTTTTTGTCA, hsa‐miR‐150‐5p: forward primer: CTCCCAACCCTTGTACCA, reverse primer: GGTCCAGTTTTTTTTTTTTTTTCACT.

### 2.6 Protein extraction and Western blot

The tissues and cells prepared for Western blot were lysed in RIPA buffer (50 mmol/L Tris‐Cl, 150 mmol/L NaCl, 1% Nonidet P‐40, 0.5% sodium deoxycholate, 1% SDS and 2 mmol/L EDTA, pH = 8.0) with protease inhibitors. The concentration of protein samples was examined with a BCA kit (Thermo Scientific, Waltham, MA). Proteins were separated by SDS‐PAGE and transferred to nitrocellulose membrane. The membranes were firstly blocked with 5% nonfat milk in TBST for 1 hour at room temperature and probed with the indicated primary antibody at 4°C overnight. The membranes were then washed by TBST for four times and incubated with peroxidase‐conjugated secondary antibodies at room temperature for 1 hour. The membranes were washed with TBST for four times and detected by ECL (Pierce, Waltham, MA).

### 2.7 Cell viability assay

Transfected cells were counted and seeded into 96‐well plates at a density of 2000 cells per well. After cultured for indicated time, the cells were changed with 100 μL fresh medium containing 10 μL CCK‐8 reagent (Beyotime, Haimen, China) and 90 μL complete medium. The absorbance at the wavelength of 450 nm was finally examined after 1 hour incubation. Data were collected for 5 days and three replicate wells were set in each group.

### 2.8 In vivo tumour models

TC‐07 or TC‐13 cells (1 × 10^6^ cells/mouse) were implanted subcutaneously on the axilla of 6‐week‐old female athymic mice provided by SLAC (Shanghai, China). All protocols involved into the animal experiments were approved by the Shanghai Tenth People's Hospital, Tongji University School of Medicine Animal Care and Use Committee. For the in vivo therapy response analysis, 20 mice implanted with either TC‐07 cells or TC‐13 cells for each group were randomly assigned to vehicle and sorafenib treatment, with sorafenib administration by intravenous injection at 20 mg/kg, with once daily administration for 20 days. To investigate the therapeutic efficacy of the combination of sorafenib with miR‐124/506 or EPZ‐6438, 20 mice implanted with TC‐13 cells for each group were randomly assigned to sorafenib plus miR‐124/506 treatment and sorafenib plus EPZ‐6438 treatment, with EPZ‐6438 administration by intravenous injection at 200 mg/kg (0.5% CMCNa + 0.1% Tween80 + ddH_2_O), with once daily administration for 20 days. Tumour growth and the survival of the mice were monitored daily for a total of 250 days.

### 2.9 Statistical analysis

The Kaplan‐Meier estimator and GraphPad Prism 6 were used to generate the survival curve. Differences between survival plots were calculated using a log‐rank test. Other experiments in this study were analysed by the Student's *t* test for the significance. *P* < 0.05 were considered to be statistically significant. ***P < 0.001, **P < 0.01, *P < 0.05.

## 3 RESULTS

### 3.1 No significant difference is observed for the expression levels of RAF and VEGFR in the thyroid tumour tissues from sorafenib sensitive and resistant patients

**Figure 1:**
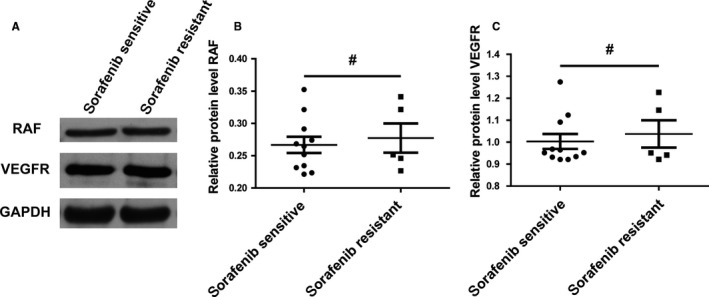
No significant difference is observed for the expression levels of RAF and VEGFR in the thyroid tumour tissues from sorafenib sensitive and resistant patients. A, RAF and VEGFP protein levels showed no difference in sorafenib sensitive and sorafenib resistant tumor tissues. Tumour tissues collected from 16 patients were divided into sorafenib sensitive group containing 11 patients and sorafenib resistant group including five patients. A Western blot was performed to detect the RAF and VEGFP protein levels of the lysates of tumor tissues. GAPDH was used as the loading control. B, No significant expression difference of RAF mRNA levels was observed between sorafenib sensitive and resistant tissues. A paired Student's *t* test was examined to compare the mRNA expression levels of RAF of the 16 patients. # indicated *P* > 0.05. C, There is no significant difference of VEGFR mRNA levels between groups of sorafenib sensitive and resistant tissues. A paired Student's *t* test was used to analysis the difference of VEGFR mRNA levels in the two groups: sorafenib sensitive group and sorafenib resistant group. # indicated *P* > 0.05

To compare the expression levels of RAF and VEGFR in the thyroid carcinoma tissues from sorafenib sensitive and resistant patients, tumour samples from a total of 16 patients with thyroid carcinoma were collected, including 11 sorafenib sensitive patients and five sorafenib resistant patients. A Western blot assay demonstrated that there is no significant difference for the expression levels of RAF and VEGFR between the tumour cells derived from sorafenib sensitive patients and tumour cells derived from sorafenib resistant patients (Figure 1A). To further validate the expression patterns of RAF and VEGFR between the sorafenib sensitive and resistant tissues, a qRT‐PCR assay was performed. The result demonstrated that the mRNA level of RAF showed no statistical difference between the sorafenib sensitive and resistant tumour tissues (Figure 1B). The same result was observed for the VEGFR expression at the mRNA level in the tumour tissues (Figure 1C). Taken together, all these data illustrate that there is no significant difference for the expression levels of RAF and VEGFR in the thyroid tumour tissues from sorafenib sensitive and resistant patients.

### 3.2 Sorafenib inhibits the proliferation ability of tumour cells derived from sorafenib sensitive patients not the sorafenib resistant patients

**Figure 2:**
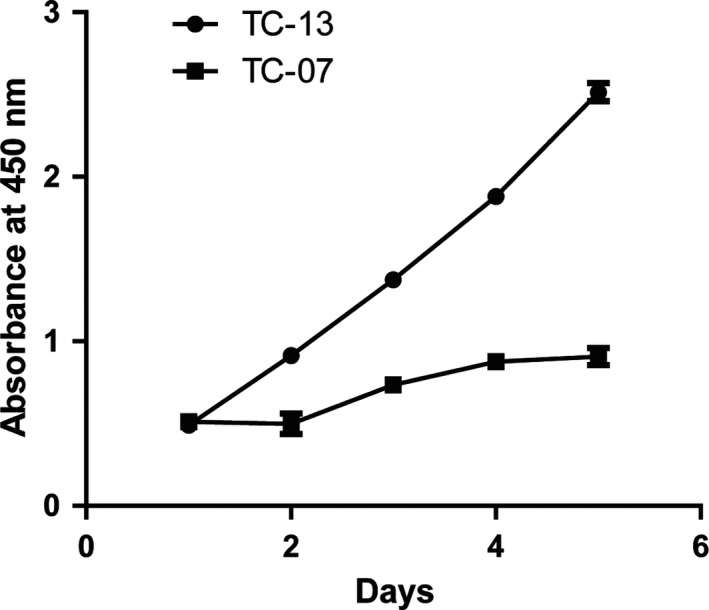
Sorafenib inhibits the proliferation ability of tumour cells derived from sorafenib sensitive patients not the sorafenib resistant patients. Several tumour cell lines were derived from the tumour tissues of the 16 patients with thyroid carcinoma. Among this, a tumor cell line named TC‐07 was established from the tumour tissues of the sorafenib sensitive patient. At the same time, we also generated a tumour cell line from the tumour tissues of the sorafenib resistant patients and named it as TC‐13. Cells were seed into 96‐well plates and treated with 10 mmol/L sorafenib for 5 d. The cell viability was detected every day by CCK‐8 kit and represented as absorbent values at 450 nm. Results are represented as mean ± SD from three independent repeats

To verify whether the thyroid carcinoma cell lines derived from sorafenib resistant patients still showed insensitivity to the sorafenib treatment, we established several tumour cell lines from the thyroid tumour tissues of the 16 patients with thyroid carcinoma. Tumour cell line generated from the tumour tissues of the sorafenib sensitive patient was named as TC‐07 and tumour cell lines obtained from the tumour tissues of the sorafenib resistant patients was named as TC‐13. A CCK assay was performed to examine the cell viability of the cell lines derived from different patients with 10 mmol/L sorafenib treatment. The results showed that sorafenib suppressed the proliferation ability of tumour cells derived from sorafenib sensitive patients not the sorafenib resistant patients (Figure 2). The sorafenib treatment experiment leads us to the conclusion that the tumour cell lines derived from tumour tissues show the same sorafenib response as the tumour tissue providers.

### 3.3 The miR‐124‐3p and miR‐506‐3p were down‐regulated in the serum from sorafenib resistant patients compared with the sensitive ones

**Figure 3:**
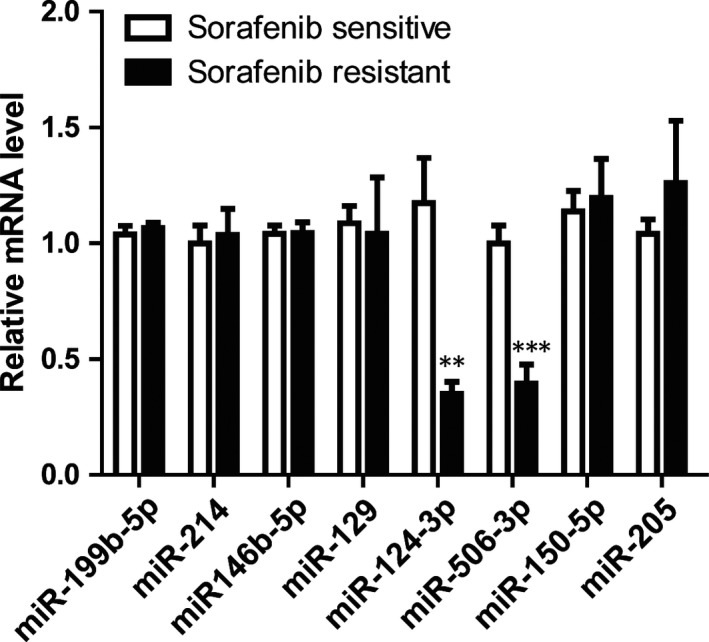
The miR‐124‐3p and miR‐506‐3p were down‐regulated in the serum from sorafenib resistant patients compared with the sensitive ones. Relative mRNA levels of miRNAs involved into the thyroid carcinoma were examined using a quantitative real‐time PCR from the serum collected from the sorafenib sensitive or resistant patients. The results represent the mean ± SD from three independent experiments. ***P < 0.001, **P < 0.01

To find out the detailed mechanism of sorafenib resistance, we examined several miRNAs with a qRT‐PCR in the serum of the patients, which were reported to play important roles during the development of thyroid cancer. The results indicated that the miR‐124‐3p and miR‐506‐3p were substantially decreased in the serum from sorafenib resistant patients compared with the sensitive ones. However, other miRNAs, including miR‐199b‐5p, miR‐214, miR146b‐5p, miR‐129; miR‐150‐5p and miR‐205, were scarcely changed between the two types of serums from resistant and sensitive patients (Figure 3). This data suggests that the resistance of sorafenib in patients may be through the regulation of miR‐124/506.

### 3.4 EZH2 was modulated by miR‐124‐3p and miR‐506‐3p in sorafenib resistant cells

**Figure 4:**
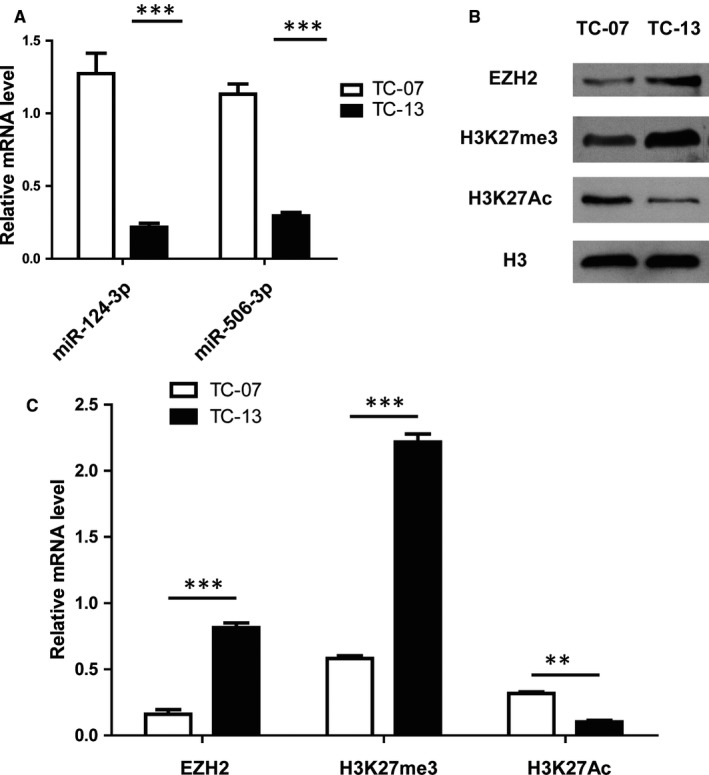
Enhancer of zeste homolog 2 (EZH2) was modulated by miR‐124‐3p and miR‐506‐3p in sorafenib resistant cells. A, The expression levels of miR‐124‐3p and miR‐506‐3p were decreased in TC‐13 cells compared with the TC‐07 cells. The mRNA levels of miR‐124‐3p and miR‐506‐3p were determined by a qRT‐PCR. The results represent the mean ± SD from three independent experiments. ***P < 0.001. B, The protein levels of EZH2 and H3K27me3 was up‐regulated, whereas, the H3K27Ac was down‐regulated in TC‐13 cells compared with the TC‐07 cells. A Western blot of cell lysates of TC‐13 and TC‐07 cell was performed to examine the protein levels of EZH2, H3K27me3 and H3K27Ac. H3 was used as the loading control. C, The relative mRNA levels of EZH2 and H3K27me3 were increased and H3K27Ac was decreased in the TC‐13 cells compared with the TC‐07 cells. The mRNA levels of EZH2, H3K27me3 and H3K27Ac were tested by a qRT‐PCR. The results represent the mean ± SD from three independent experiments. ***P < 0.001, **P < 0.05

To further prove that the expression levels of miR‐124/506 had relationship with the resistance of sorafenib, we examined the mRNA levels of miR‐124 and miR‐506 with a qRT‐PCR assay. The results showed that both of the miR‐124 and miR‐506 were remarkably decreased in TC‐13 cells derived from the sorafenib resistant patient, compared with TC‐07 cells established from the sorafenib sensitive patient (Figure 4A). Interestingly, a bioinformatics analysis predicted that miR‐124 and miR‐506 shared a common target gene, EZH2. A further Western blot result put evidence on this prediction that the protein level of EZH2 was significantly highly expressed in the TC‐13 cells compared with the TC‐07 cells (Figure 4B). Subsequently, the expression level of H3K27me3 was up‐regulated, whereas, the H3K27Ac was down‐regulated in TC‐13 cells compared with the TC‐07 cells (Figure 4B). Consistently, the relative mRNA levels of EZH2 and H3K27me3 were markedly elevated and H3K27Ac was dramatically reduced in the TC‐13 cells compared with the TC‐07 cells (Figure 4C). In summary, all these data indicate that the EZH2 was modulated by miR‐124/506 in sorafenib resistant cells.

### 3.5 Targeting EZH2 by overexpression of miR‐124/506 or EPZ‐6438 treatment inhibits the proliferation ability of sorafenib resistant thyroid tumour cells via epigenetic regulation

**Figure 5:**
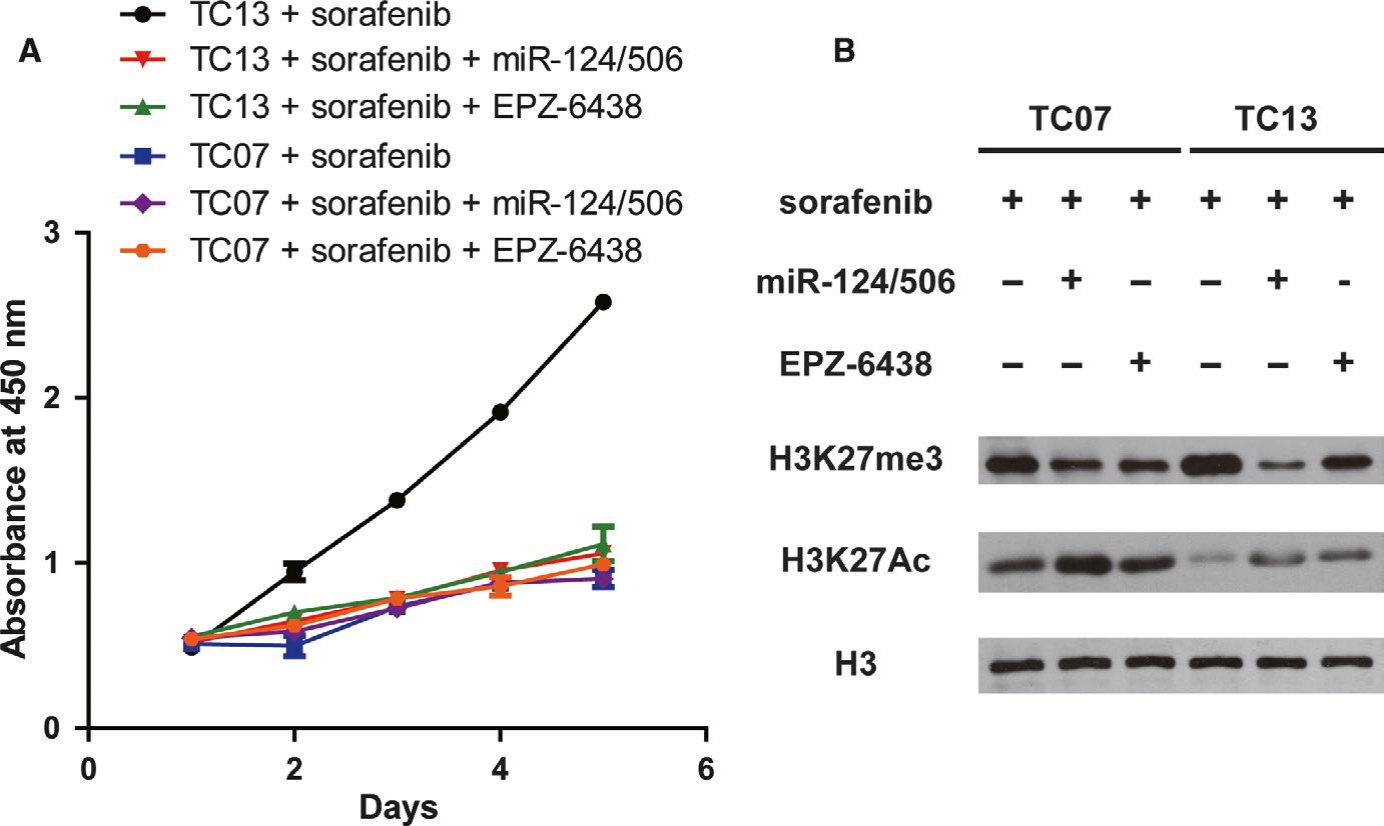
Targeting enhancer of zeste homolog 2 by overexpression of miR‐124/506 or EPZ‐6438 treatment inhibits the proliferation ability of sorafenib resistant thyroid tumor cells via epigenetic regulation. A, The proliferation ability of TC‐13 cells was inhibited when cells were overexpressed with miR‐124/506 or treated with 1 μmol/L EPZ‐6438. The cell viability was examined for 5 d by CCK‐8 kit and the absorbance results at 450 nm were represented as mean ± SD from three independent repeats. B, Overexpression of miR‐124/506 or EPZ‐6438 treatment promotes the protein expression of H3K27me3 and suppresses the protein level of H3K27Ac in thyroid tumour cells. A Western blot was performed to determine the protein levels of H3K27me3 and H3K27Ac with indicated antibodies. H3 was tested as a loading control

To identify whether the regulation of sorafenib resistance by miR‐124/506 through targeting of EZH2 directly, we modulated the EZH2 levels by miR‐124/506 overexpression or EPZ‐6438 (an EZH2 inhibitor) treatment. The results demonstrated that either exogenous overexpression of miR‐124/506 or EPZ‐6438 treatment effectively enhanced the suppression ability of sorafenib on the proliferation of TC‐13 cells (Figure 5A). We next examined the protein expression levels of H3K27me3 and H3K27Ac in the miR‐124/506 overexpressed or EPZ‐6438 treated thyroid tumour cells by Western blot. The data indicated that the up‐regulation of EZH2 remarkably promoted the protein expression of H3K27me3 and suppressed the protein level of H3K27Ac (Figure 5B). Taken together, all these data lead to the conclusion that targeting EZH2 by overexpression of miR‐124/506 or EPZ‐6438 treatment inhibits the proliferation ability of sorafenib resistant thyroid tumour cells via epigenetic regulation.

### 3.6 Combination of sorafenib with miR‐124/506 overexpression or EZH2 inhibitor improves the survival in mice model

**Figure 6:**
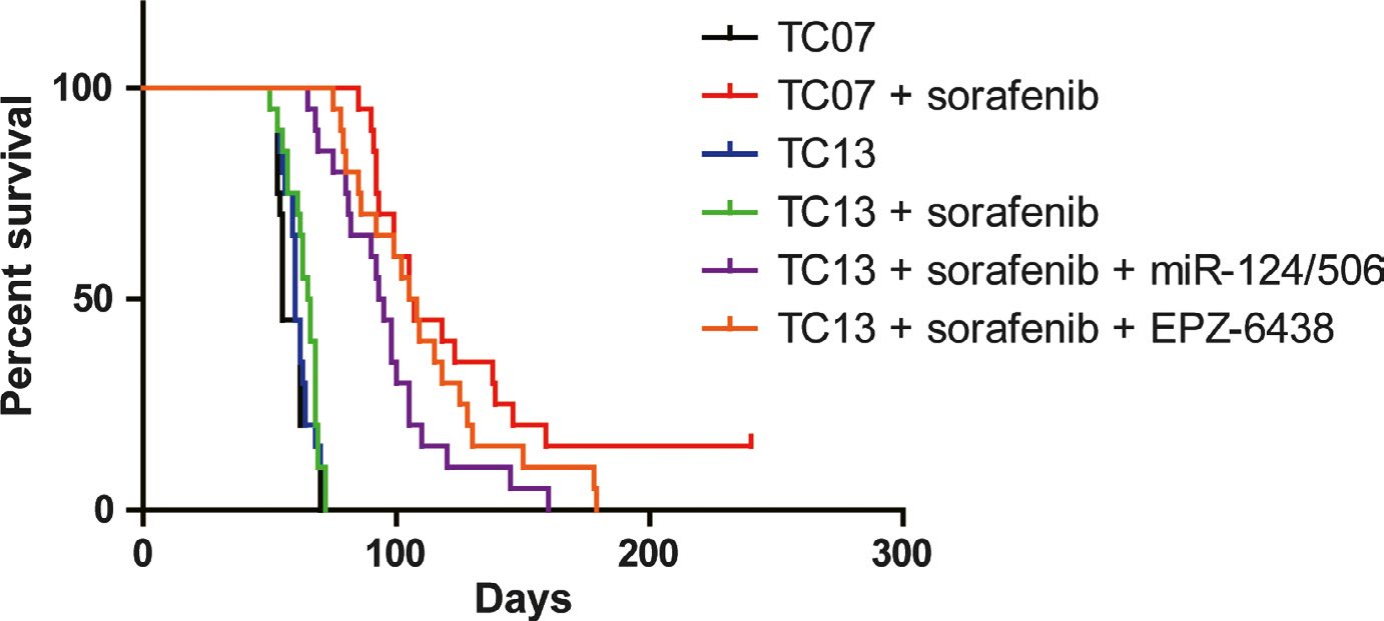
Combination of sorafenib with miR‐124/506 overexpression or enhancer of zeste homolog 2 inhibitor improves the survival in mice model. Survival curve of the mice were injected with TC‐07 or TC‐13 cells (1 × 10^6^ cells/mouse) by subaxillary inoculation. The sorafenib was given every day for 20 d by 20 mg/kg with intravenous injection or a combination with miR‐124/506 mimics or the amount of EPZ‐6438 by 200 mg/kg. Each group has 20 mice

We next investigated whether miR‐124/506 overexpression or EZH2 inhibitor had any effect on the tumour formation in vivo. We firstly established an animal model with subaxillary inoculation of TC‐07 or TC‐13 cells and then, the sorafenib was given every day for 20 days by 20 mg/kg with intravenous injection or a combination with miR‐124/506 mimics or the amount of EPZ‐6438 by 200 mg/kg. The combination of sorafenib with miR‐124/506 mimics or EZH2 inhibitor significantly improved the survival in mice model (Figure 6). The result indicates that modulating EZH2 expression by miR‐124/506 mimics or EPZ‐6438 treatment may be a new strategy for the improvement of thyroid carcinoma therapy for the sorafenib insensitive patients.

## 4 DISCUSSION

Thyroid carcinoma is the most common and worldwide endocrine malignancy, the incidence of which has been rapidly increasing all over the world.^1^, ^3^, ^4^ Although most of the patients with thyroid carcinoma felt in remission following therapies, the recurrence rate still remains high, leading to a decreased survival rate.^7^ Sorafenib was a well‐known inhibitor targeting RAF, RTK, VEGFR and REK signalling pathways for the therapy of multiple cancers.^9^ Even tough emerging studies have reported the therapeutic effects for the DTC by sorafenib treatment, the side effects and drug resistance are still the big problems remained to be solved.^9^ Therefore, the detailed mechanism and new therapeutic methods are still needed to be further explored. In this study, we aim to investigate novel target to broaden the choices for thyroid carcinoma therapy.

In this study, we firstly found that some of the patients with thyroid carcinoma showed insensitivity for the sorafenib treatment. Further Western blot and qRT‐PCR experiments demonstrated that the expression levels of sorafenib targets, RAF and VEGFR, are scarcely changed between the sorafenib resistant and sensitive tumour tissues and tumour cell lines. To investigate the potential mechanisms for the sorafenib resistance in patients, we examined the expression patterns of miRNAs reported to play essential roles in the thyroid carcinoma development. Our results indicated that the miR‐124 and miR‐506 were substantially reduced in the serum collected from sorafenib resistant patients. Consistently, the same results were observed in the thyroid carcinoma cell lines derived from patients with thyroid carcinoma. Interestingly, the results from bioinformatics analysis and Western blot assay indicated that miR‐124 and miR‐506 shared the same target gene, EZH2, a subunit of PRC2. Considering that the EZH2 has been well studied as a histone modification regulation factor previously, we examined the protein and mRNA levels of H3K27me3 and H3K27Ac. Encouragingly, we found that the intervention of EZH2 by miR‐124/506 overexpression or EPZ‐6438 treatment led to the down‐regulated H3K27me3 and up‐regulated H3K27Ac. Moreover, the sorafenib resistant tumour cells regained sensitivity by the combined treatment of sorafenib with EZH2 intervention, which might be helpful for the better understanding of the molecule mechanism of thyroid cancer tumorigenesis and for investigating suitable novel target for thyroid carcinoma therapy.

The sorafenib treatment is already widely used for the therapy of DTC with promising preclinical studies.^9^ However, few researches were reported for its side effects and drug resistance. In our study, we found that some patients showed resistance to sorafenib and no significant expression difference of sorafenib targeted genes was overserved, which prompts us to seek new strategies to choose for the DTC therapy with sorafenib resistance.

The dysregulation of EZH2 in tumour cells contributes to inhibitory roles of tumour suppressor genes, leading to the carcinogenesis.^26^ The miR‐124‐3p and miR‐506‐3p were reported as potential biomarker for the DTC.^18^, ^19^ In our study, we found that overexpression of miR‐124‐3p and miR‐506‐3p modulated the expression of EZH2 in sorafenib resistant thyroid cancer cells, leading to the regulation of H3K27me3 and H3K27Ac. To the best of our knowledge, this is the first report explaining the sorafenib resistance by epigenetic regulation through EZH2 intervention. However, despite of these reported miRNAs involved into the thyroid tumorigenesis, we could not exclude other possible factors playing essential regulatory roles in thyroid carcinoma therapy for the resistance of sorafenib, which needs more studies.

Emerging studies have revealed that the miR‐124 could directly target the EZH2 and suppress its expression in various cancers, including cholangiocarcinoma, prostate cancer, gastric cancer and hepatocellular carcinoma.^27^, ^28^, ^29^, ^30^, ^31^ Moreover, miR‐506 was reported to inhibit tumour cell proliferation and metastasis in colon cancer by directly targeting EZH2.^32^ In our study, we demonstrated that miR‐124/506 mimics modulated EZH2 expression to help sorafenib resistant thyroid tumour cells regained the sensitivity for sorafenib treatment. Taken together, our findings put evidence that miR124/506 have the ability to regulate EZH2, leading to the decreased H3K27me3 and increased H3K27Ac, which was mirrored by previous research findings.

However, there still exist some limitations for this research. According to the research conditions in the hospital, the main limitation involved into this study is that the number of clinical samples in this study is relatively small, leading to the difficulties for the credibility and generalization of the experimental result. Therefore, further research should be conducted with more clinical samples to reveal the detail mechanism of sorafenib resistance in some of patients with thyroid carcinoma.

In summary, we found out that sorafenib was not suitable for all of the thyroid patients and revealed the reasons why they responded differently to sorafenib treatment, which was the abnormal regulation of PRC signalling pathway. Furthermore, we demonstrated that the combined treatment of sorafenib with miR‐124/506 mimics or EZH2 inhibitor could efficiently improve the sensitivity of sorafenib resistant thyroid tumour cells for sorafenib treatment in vitro and in vivo. Clinically, the levels of EZH2 or miR‐124/506 should be detected before the treatment of sorafenib. The EZH2 inhibitor or miR‐124/506 mimics should be used with the combination of sorafenib to improve the therapy effects when patients showed abnormal expression of EZH2 or miR‐124/506. Taken together, our findings suggested a potential role of EZH2 in thyroid carcinoma therapy.

## 5 CONCLUSION

1. Sorafenib is not suitable for all of the thyroid patients and the reason is abnormal regulation of PRC signalling pathway.
2. The combined treatment of sorafenib with miR‐124/506 mimics or EZH2 inhibitor could efficiently improve the sensitivity of sorafenib resistant thyroid tumour cells for sorafenib treatment in vitro and in vivo.
3. Our findings suggested a potential therapeutic role of EZH2 in thyroid carcinoma therapy.

## AUTHOR CONTRIBUTIONS

Z.W., J.D., J.Y. and Y.Z. conducted the experiments; Z.W. and J.D. analyzed the data; Z.W. and Z.Y. wrote the manuscript; Z.Y. conceived and coordinated the study.

## DISCLOSURE

The authors declare that they have no conflict of interest.

## Data Availability

Data could be obtained upon request to the corresponding author.
